# Supplementary material for: Chromatin remodeller Fun30Fft3 induces nucleosome disassembly to facilitate RNA polymerase II elongation
Source: Nat Commun. 2017 Feb 20;8:14527. doi: 10.1038/ncomms14527 (PMC5321744; doi:10.1038/ncomms14527)
Supplement: Supplementary Information — Supplementary Figures and Supplementary Tables [file ncomms14527-s1.pdf]

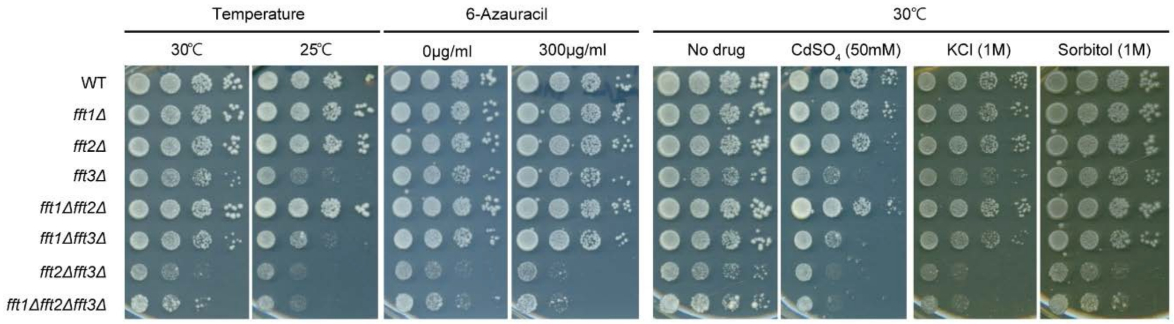

Supplementary Figure 1. Fun30 paralogs Fun30^{Fft2} and Fun30^{Fft3} are redundant in fission yeast. Spotting assays were used to assess genetic interactions among Fun30 paralogs in fission yeast. Low temperature (25℃; mild cold shock condition), 6-Azauracil (blocking growth in transcription elongation defective mutant cells), cadmium sulfate (causing oxidative stress), potassium chloride and sorbitol (causing hyper-osmotic shock) were used to clarify the genetic interaction between Fun30 paralogs mutants.

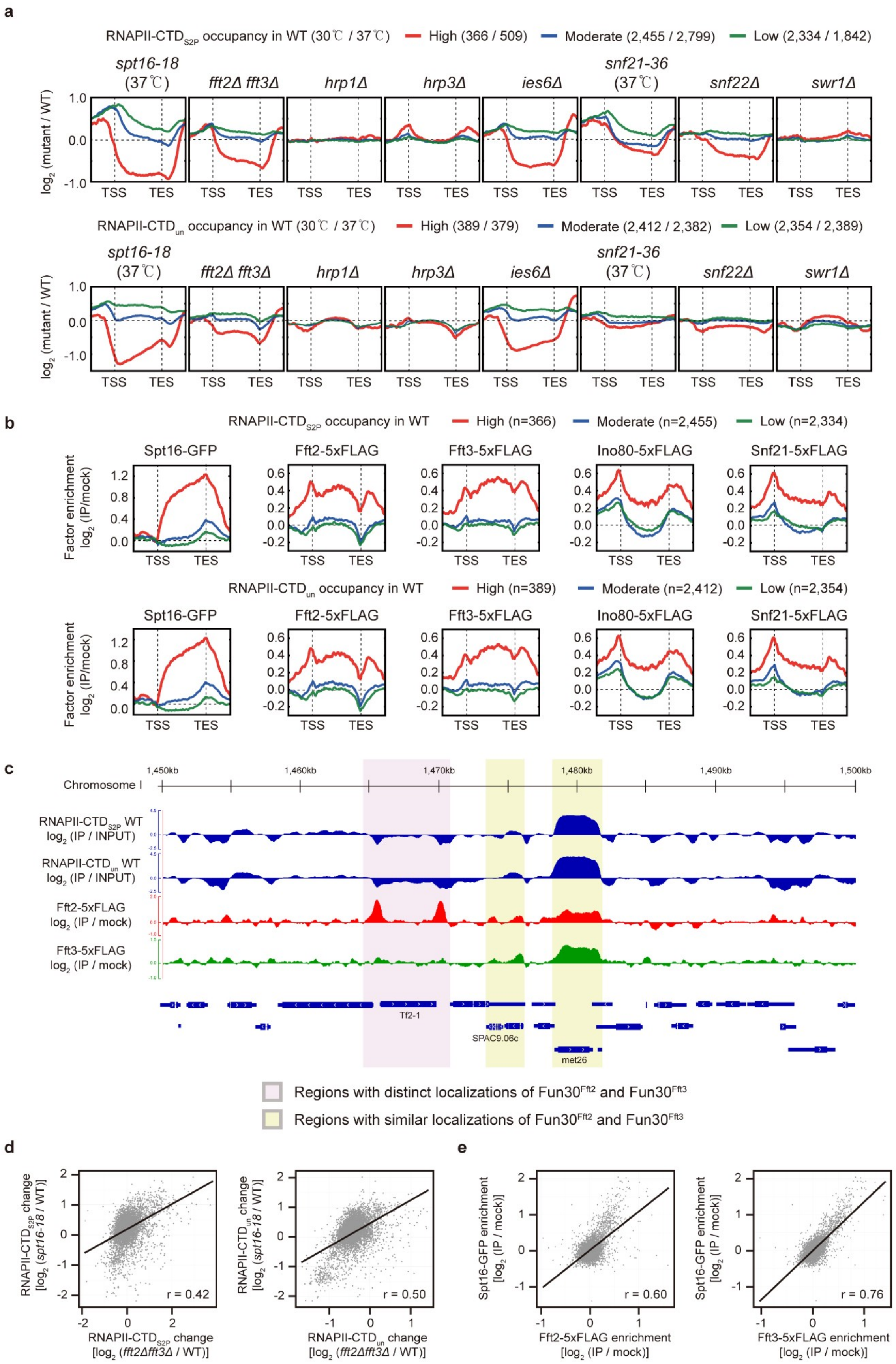
a
RNAPII-CTD$_{S2P}$ occupancy in WT (30℃ / 37℃)
High (366 / 509)
Moderate (2,455 / 2,799)
Low (2,334 / 1,842)
spt16-18 (37℃)
fft2Δ fft3Δ
hrp1Δ
hrp3Δ
ies6Δ
snf21-36 (37℃)
snf22Δ
swr1Δ
log$_2$ (mutant / WT)
1.0
0.0
-1.0
TSS
TES
RNAPII-CTD$_{un}$ occupancy in WT (30℃ / 37℃)
High (389 / 379)
Moderate (2,412 / 2,382)
Low (2,354 / 2,389)
b
RNAPII-CTD$_{S2P}$ occupancy in WT
High (n=366)
Moderate (n=2,455)
Low (n=2,334)
Spt16-GFP
Fft2-5xFLAG
Fft3-5xFLAG
Ino80-5xFLAG
Snf21-5xFLAG
Factor enrichment log$_2$ (IP/mock)
1.2
0.8
0.4
0.0
0.6
0.4
0.2
0.0
-0.2
TSS
TES
RNAPII-CTD$_{un}$ occupancy in WT
High (n=389)
Moderate (n=2,412)
Low (n=2,354)
c
Chromosome I
1,450kb
1,460kb
1,470kb
1,480kb
1,490kb
1,500kb
RNAPII-CTD$_{S2P}$ WT log$_2$ (IP / INPUT)
RNAPII-CTD$_{un}$ WT log$_2$ (IP / INPUT)
Fft2-5xFLAG log$_2$ (IP / mock)
Fft3-5xFLAG log$_2$ (IP / mock)
Tf2-1
SPAC9.06c
met26
Regions with distinct localizations of Fun30^{Fft2} and Fun30^{Fft3}
Regions with similar localizations of Fun30^{Fft2} and Fun30^{Fft3}
d
RNAPII-CTD$_{S2P}$ change [log$_2$ (spt16-18 / WT)]
RNAPII-CTD$_{S2P}$ change [log$_2$ (fft2Δfft3Δ / WT)]
r = 0.42
RNAPII-CTD$_{un}$ change [log$_2$ (spt16-18 / WT)]
RNAPII-CTD$_{un}$ change [log$_2$ (fft2Δfft3Δ / WT)]
r = 0.50
e
Spt16-GFP enrichment [log$_2$ (IP / mock)]
Fft2-5xFLAG enrichment [log$_2$ (IP / mock)]
r = 0.60
Fft3-5xFLAG enrichment [log$_2$ (IP / mock)]
r = 0.76

Supplementary Figure 2. RNAPII occupancy changes in FACT and chromatin remodeler mutants, and the gene occupancies of FACT and selected chromatin remodelers. (**a, b**) The data and analyses are as described for Figure 1a,c, except that the profiles were generated for total RNAPII genes clustered into three groups (high, moderate and low) according to their occupancies of RNAPII-CTD_{S2P} (up) and RNAPII-CTD_{un} (down). Regions upstream of the TSS and downstream of the TES (0.5 kb) were used as intergenic regions near each metagene. (**c**) Genome-browser view of a representative chromosome region, showing the different binding pattern between Fft2-5xFLAG (red) and Fft3-5xFLAG (green). Regions where both $Fun30^{Fft2}$ and $Fun30^{Fft3}$ are co-localized (yellow shaded) and regions where $Fun30^{Fft2}$ is distinctively enriched (purple shaded) were highlighted. (**d**) Scatterplot analyses to describe the similarity of RNAPII-CTD_{S2P} (left) and RNAPII-CTD_{un} (right) occupancy change in *fft2Δ fft3Δ* cells and *spt16-18* cells. RNAPII occupancies (IP/INPUT) at ORFs of 5,150 protein-coding genes were calculated by using merged reads obtained from biological duplicates for wild-type and *spt16-18* cells except *fft2Δ fft3Δ* cells for which sequence reads from single sample were used. Correlation coefficients (r) were calculated by the Pearson method. The r values were 0.42 and 0.5 for the comparison of RNAPII-CTD_{S2P} and RNAPII-CTD_{un} occupancy changes in *fft2Δ fft3Δ* cells and *spt16-18* cells. (**e**) Scatterplot analyses to show the similarity of factor enrichment level between Fun30 paralogs (left: $Fun30^{Fft2}$, right: $Fun30^{Fft3}$) and Spt16. The ChIP enrichments (IP/mock) at ORFs of 5,150 protein-coding genes were calculated by using merged reads obtained from biological duplicates. Correlation coefficients (r) were calculated according to the Pearson method. The r values were 0.60 and 0.76 for the factor enrichment comparison for Spt16-GFP with Fft2-5xFLAG and Fft3-5xFLAG.

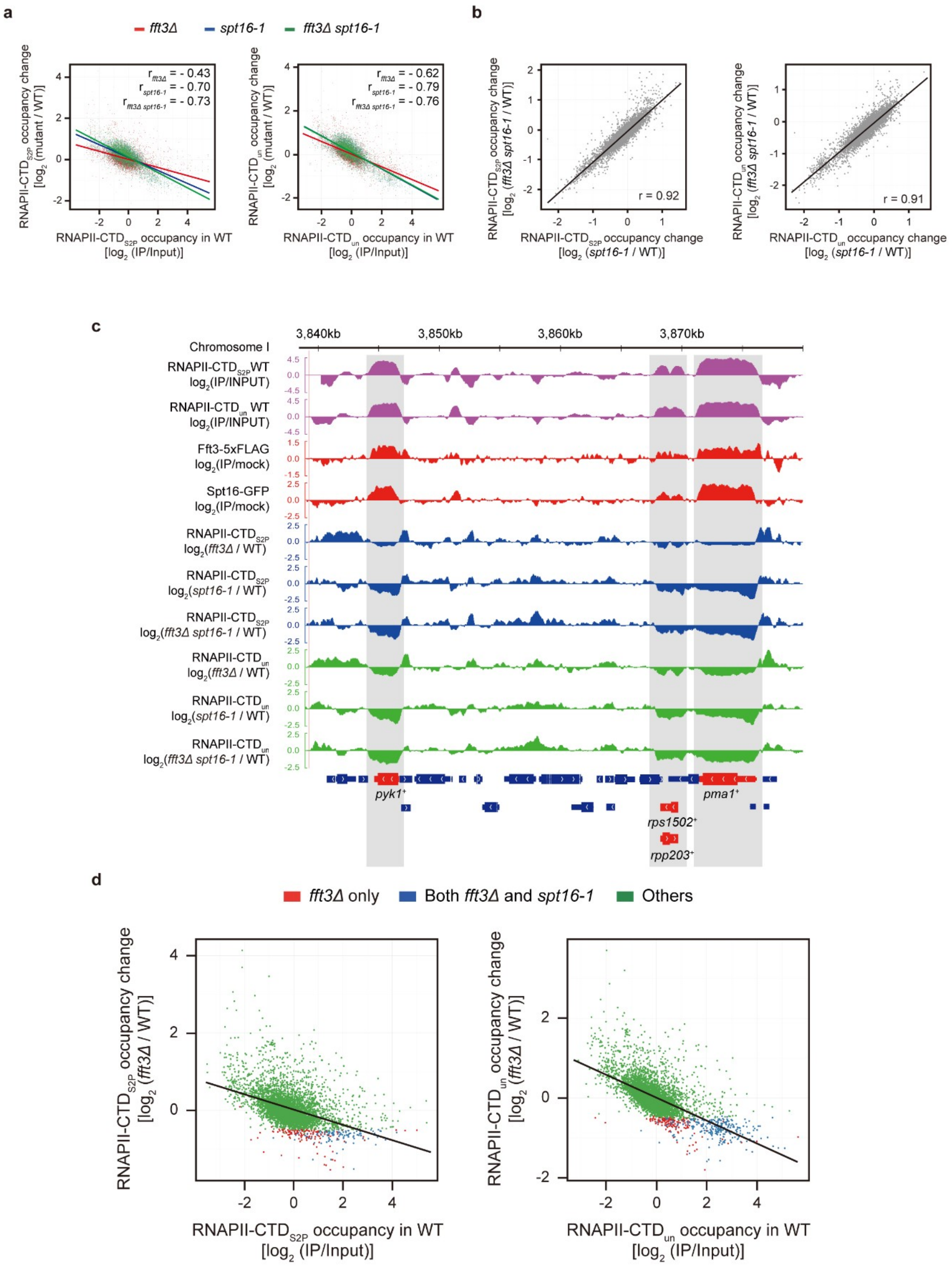

a
fft3Δ
spt16-1
fft3Δ spt16-1
RNAPII-CTD$_{S2P}$ occupancy change [log$_2$ (mutant / WT)]
RNAPII-CTD$_{S2P}$ occupancy in WT [log$_2$ (IP/Input)]
$r_{fft3Δ}$ = - 0.43
$r_{spt16-1}$ = - 0.70
$r_{fft3Δ\ spt16-1}$ = - 0.73
RNAPII-CTD$_{un}$ occupancy change [log$_2$ (mutant / WT)]
RNAPII-CTD$_{un}$ occupancy in WT [log$_2$ (IP/Input)]
$r_{fft3Δ}$ = - 0.62
$r_{spt16-1}$ = - 0.79
$r_{fft3Δ\ spt16-1}$ = - 0.76
b
RNAPII-CTD$_{S2P}$ occupancy change [log$_2$ (fft3Δ spt16-1 / WT)]
RNAPII-CTD$_{S2P}$ occupancy change [log$_2$ (spt16-1 / WT)]
r = 0.92
RNAPII-CTD$_{un}$ occupancy change [log$_2$ (fft3Δ spt16-1 / WT)]
RNAPII-CTD$_{un}$ occupancy change [log$_2$ (spt16-1 / WT)]
r = 0.91
c
3,840kb
3,850kb
3,860kb
3,870kb
Chromosome I
RNAPII-CTD$_{S2P}$ WT log$_2$(IP/INPUT)
RNAPII-CTD$_{un}$ WT log$_2$(IP/INPUT)
Fft3-5xFLAG log$_2$(IP/mock)
Spt16-GFP log$_2$(IP/mock)
RNAPII-CTD$_{S2P}$ log$_2$(fft3Δ / WT)
RNAPII-CTD$_{S2P}$ log$_2$(spt16-1 / WT)
RNAPII-CTD$_{S2P}$ log$_2$(fft3Δ spt16-1 / WT)
RNAPII-CTD$_{un}$ log$_2$(fft3Δ / WT)
RNAPII-CTD$_{un}$ log$_2$(spt16-1 / WT)
RNAPII-CTD$_{un}$ log$_2$(fft3Δ spt16-1 / WT)
pyk1+
pma1+
rps1502+
rpp203+
d
fft3Δ only
Both fft3Δ and spt16-1
Others
RNAPII-CTD$_{S2P}$ occupancy change [log$_2$ (fft3Δ / WT)]
RNAPII-CTD$_{S2P}$ occupancy in WT [log$_2$ (IP/Input)]
RNAPII-CTD$_{un}$ occupancy change [log$_2$ (fft3Δ / WT)]
RNAPII-CTD$_{un}$ occupancy in WT [log$_2$ (IP/Input)]

Supplementary Figure 3. The function of Fun30^{Fft3} in regulating RNAPII occupancy at transcribing regions largely overlaps with that of FACT. (**a**) Merged scatterplots show transcription-dependent decreases in the occupancies of RNAPII-CTD$_{S2P}$ (left) and RNAPII-CTD$_{un}$ (right) in *fft3Δ* (red), *spt16-1* (blue), and *fft3Δ spt16-1* (green) cells. The correlation coefficients were calculated according to the Pearson method. (**b**) Scatterplots illustrate the similarities in the occupancy changes of RNAPII-CTD$_{S2P}$ (top) and RNAPII-CTD$_{un}$ (bottom) in *spt16-1* and *fft3Δ spt16-1* cells. RNAPII occupancies (IP/INPUT) at ORFs of 5,150 protein-coding genes were calculated by using merged reads obtained from biological duplicates. Correlation coefficients (r) were calculated by the Pearson method. The r values were 0.92 and 0.91 for the comparison of RNAPII-CTD$_{S2P}$ and RNAPII-CTD$_{un}$ occupancy changes in *spt16-1* and *fft3Δ spt16-1* cells. (**c**) Genome-browser view of a representative chromosome region, showing the similarities in RNAPII occupancy changes among *fft3Δ*, *spt16-1*, and *fft3Δ spt16-1* cells. All cells were incubated at 25ºC and then shifted to 37ºC for 2 hours, except for cells expressing Fft3-5xFLAG and Spt16-GFP, which were continuously grown at 30ºC. Transcribing regions with high RNAPII occupancy are gray shaded and the corresponding genes are red highlighted. (**d**) Scatterplots showing the relation between transcription-dependent RNAPII-CTD$_{S2P}$ (left) and RNAPII-CTD$_{un}$ (right) occupancy decrease in three gene groups – those where RNAPII occupancy maintenance was affected in *fft3Δ* cells only (red), both *fft3Δ* and *spt16-1* (blue), and the others (green) – clustered in Fig. 2b. RNAPII occupancies (IP/INPUT) at ORFs of 5,150 protein-coding genes were calculated by using merged reads obtained from biological duplicates. The black line is the trend line of the transcription-dependent RNAPII occupancy defect in *fft3Δ* which was already described in Supplementary Fig 3a.

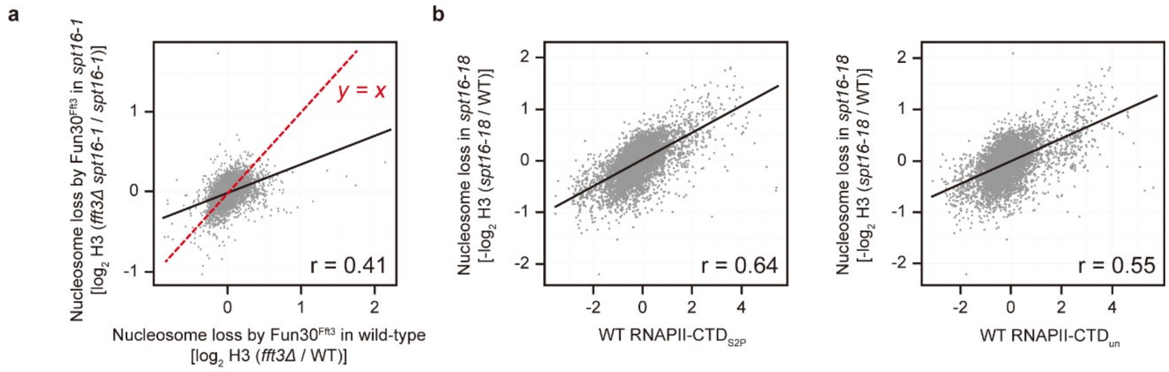

Supplementary Figure 4. **Nucleosome occupancy is determined in Spt16-dependent manner during RNAPII elongation at transcribing regions.** (**a**) Scatterplot analysis illustrates the importance of Spt16 in nucleosome loss caused by the deletion of *fft3*$^{+}$. Histone H3 occupancies (IP/INPUT) at ORFs of 5,149 protein-coding genes were calculated by using merged reads obtained from biological duplicates. Correlation coefficient (r) was calculated by the Pearson method. The r value was 0.41 for the comparison between the nucleosome loss by Fun30^{Fft3} in wild-type and that in *spt16-1* cells. (**b**) Scatterplot analyses describes RNAPII elongation-dependent nucleosome loss in *spt16-18* at transcribing regions. RNAPII and histone H3 occupancies (IP/INPUT) at ORFs of 5,150 protein-coding genes were calculated by using merged reads obtained from biological duplicates. Correlation coefficients (r) were calculated by the Pearson method. The r values were 0.64 and 0.55 for the comparison RNAPII (RNAPII-CTD$_{S2P}$ and RNAPII-CTD$_{un}$) occupancy in wild-type cells and nucleosome loss in *spt16-18* cells.

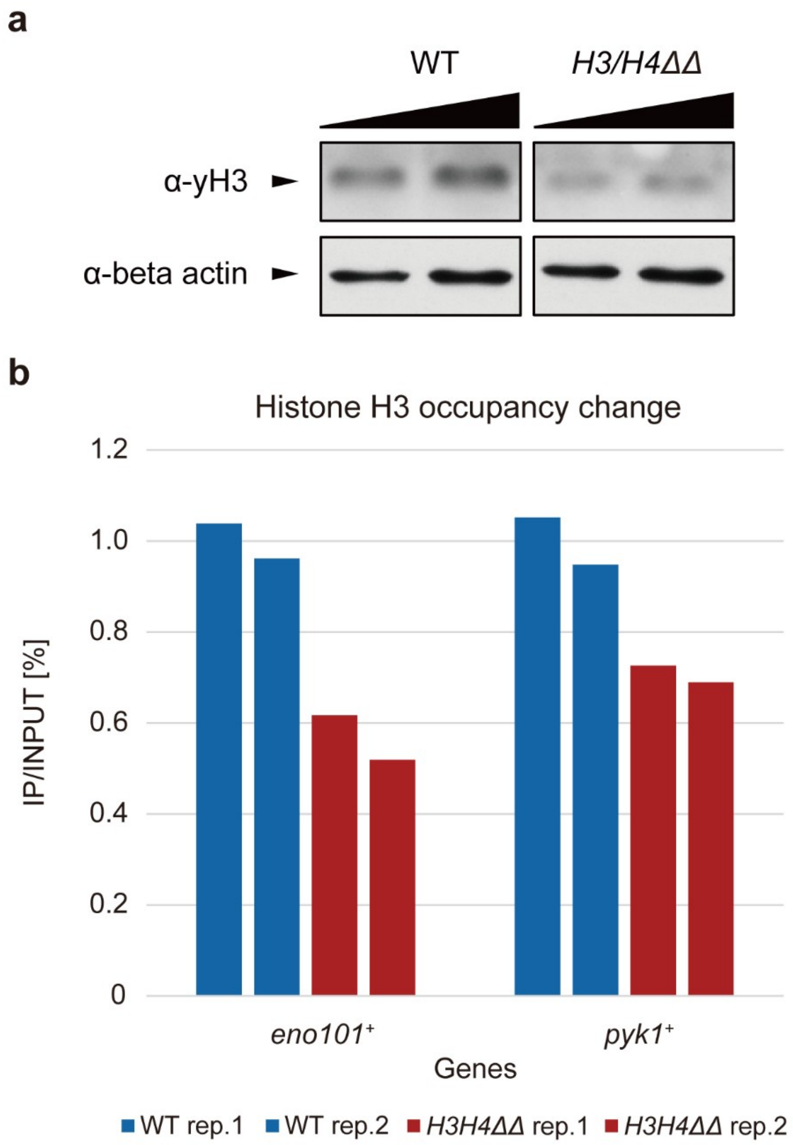

Supplementary Figure 5. **Nucleosome levels are globally reduced in cells that lack two of the three genes encoding histone H3 and H4.** (**a**) Western blot analysis was used to assess the reduction of soluble histone H3 in *H3/H4ΔΔ* cells relative to wild-type cells. β-actin was used as a loading control. (**b**) ChIP analysis shows that histone H3 occupancy is decreased in *H3/H4ΔΔ* cells at the transcribing regions of the highly transcribed genes, *eno101*$^{+}$ and *pyk1*$^{+}$. ChIP-qPCR results are presented individually for the biological replicates used in the ChIP-Seq analysis. Primer sequences are given in **Supplementary Table 3**.

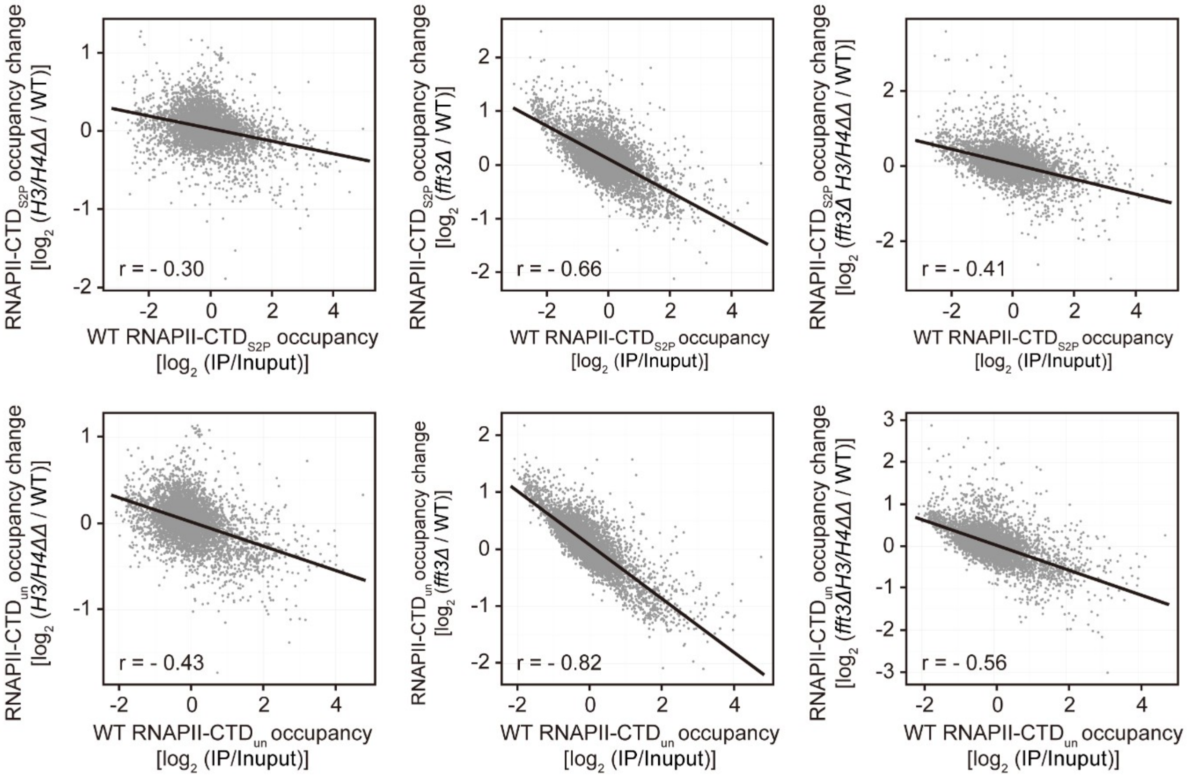

Supplementary Figure 6. **The reduction of RNAPII occupancy in *fft3Δ* cells is suppressed by the deletion of histone genes.** RNAPII occupancy change in *fft3Δ*, *H3/H4ΔΔ* and *fft3Δ H3/H4ΔΔ* cells were compared by scatterplots. RNAPII occupancies (IP/INPUT) at ORFs of 5,145 protein-coding genes were calculated by using merged reads obtained from biological duplicates. Correlation coefficients (r) were calculated by the Pearson method. The r values were -0.30, -0.66 and -0.41 for the transcription-dependent RNAPII-CTD_{S2P} occupancy change in *H3/H4ΔΔ*, *fft3Δ* and *fft3Δ H3/H4ΔΔ* cells and -0.43, -0.82 and -0.56 for the transcription-dependent RNAPII-CTD_{un} occupancy change in *H3/H4ΔΔ*, *fft3Δ* and *fft3Δ H3/H4ΔΔ* cells.

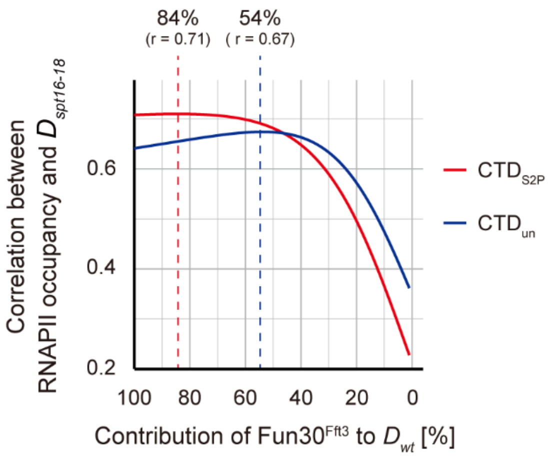

Supplementary Figure 7. Fun30^{Fft3} plays a major role in transcription-coupled nucleosome disassembly *in vivo*. The contribution of Fun30^{Fft3} to transcription-coupled nucleosome disassembly (for RNAPII-CTD$_{S2P}$ and RNAPII-CTD$_{un}$) *in vivo* was estimated by calculating the point at which RNAPII occupancy at ORFs is maximally correlated with $D_{spt16-18}$ (84% and 54% for RNAPII-CTD$_{S2P}$ and RNAPII-CTD$_{un}$, respectively). For the method of estimation, see **Methods**.

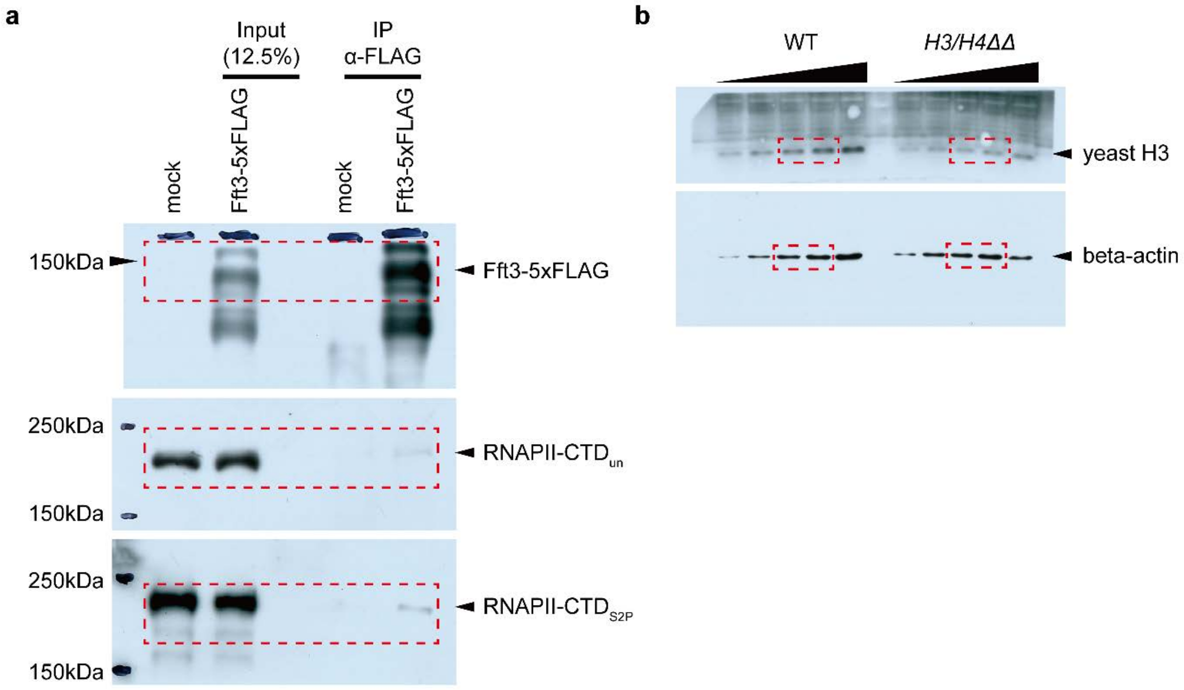

Supplementary Figure 8. Uncropped scans of all immunoblots. (**a**) Original and full blot for Fig. 3a. The cropped regions were indicated by dotted line box. (**b**) Original and full blot for Supplementary Fig. 5a. The cropped regions were indicated by dotted line box.

Supplementary Table 1. MudPIT result of Fft3 isolated by tandem affinity purification (TAP)

systematic name	standard name	Spectral Count	Sequence Coverage [%]	Length	MolWt
SPBC4C3.12	sep1	2	3	663	73032
SPCC63.02c	aah3	2	3.4	564	63206
SPBC2D10.18	abc1	2	2.1	610	68567
SPAC31G5.19	abo1	1	1.6	1190	135351
SPBP8B7.29	abz1	2	3.8	718	80598
SPBC32H8.12c	act1	249	50.1	375	41765
SPBC106.04	ada1	1	2.6	846	97455
SPBC405.01	ade1	1	1.9	788	85231
SPCPB16A4.03c	ade10	2	3.8	585	64123
SPCC1322.13	ade6	3	6.2	552	60013
SPBC14F5.09c	ade8	1	5	482	54307
SPBC2G2.08	ade9	1	3	969	105412
SPCC13B11.01	adh1	21	27.7	350	37396
SPBC30B4.03c	adn1	6	14.3	391	43837
SPBC1289.10c	adn2	2	5.1	743	81159
SPCC1494.10	adn3	5	8.6	964	104592
SPBPB21E7.07	aes1	1	6.1	296	32229
SPBC2D10.19c	alb1	1	25.7	105	11637
SPBC947.01	alf1	2	3.8	660	71733
SPAC23H4.12	alp13	1	3.9	337	39138
SPBP23A10.08	alp5	17	21.7	433	48773
SPCC1919.03c	amk2	1	5	298	32970
SPBC18H10.20c	any1	1	3.9	361	40144
SPBC1921.05	ape2	1	2.7	882	99446
SPBC947.02	apl2	9	9.2	683	76682
SPBC691.03c	apl3	2	3.4	878	100079
SPCP1E11.06	apl4	18	16.1	865	96042
SPAC23H3.06	apl6	3	3.4	745	83871
SPBP16F5.07	apm1	7	7.3	426	48956
SPBC651.11c	apm3	1	4.2	425	48422
SPAC13G6.14	aps1	1	3.8	210	23724
SPBC14C8.06	arc1	1	10.9	377	41597
SPBC1778.08c	arc3	1	21.3	174	19844
SPAC4G9.09c	arg11	1	3.6	885	97705
SPBC428.05c	arg12	2	4.6	410	46085
SPBC215.08c	arg4	2	3.8	1160	127480
SPBC56F2.09c	arg5	13	36.4	415	45662
SPAC1834.02	aro1	3	1.5	1573	173781
SPAC630.03	arp3	1	7	427	47373
SPBC365.10	arp5	1	3.2	721	82364
SPAC664.02c	arp8	7	11.1	620	70058
SPAPB1A10.09	ase1	23	11.9	731	83173
SPBC13G1.08c	ash2	3	5.5	652	74253
SPAC13G6.10c	asl1	1	3.8	530	54211
SPBC119.10	asn1	2	3.2	557	63241
SPCC1672.06c	asp1	2	1.7	920	105680
SPBC800.05c	atb2	5	6.9	449	50536
SPBC29B5.01	atf1	2	8.5	566	59711
SPCC63.08c	atg1	11	15.9	830	91585

SPAC7D4.04	atg11		1	1.6	926	106770
SPAC222.12c	atp2		7	9.5	525	56876
SPAC22F3.07c	atp20		1	12.7	118	13640
SPCC1840.06	atp5		2	10.2	216	23988
SPAC631.02	bdf2		2	5.9	769	85977
SPAC644.18c	bet3		1	6.6	183	20837
SPCC1235.02	bio2		12	12.1	363	40651
SPAC22A12.15c	bip1		24	12.4	663	73227
SPAC8F11.06	brr6		1	4.7	297	33574
SPCC330.11	btb1		1	1.5	1347	152586
SPAC4F10.14c	btf3		5	26.5	151	16202
SPAC12B10.03	bun62		1	2.6	543	61811
SPAC3A12.14	cam1		5	24.7	150	16906
SPCC306.09c	cap1		13	10.2	551	60244
SPAC29A4.04c	cbf5		4	5.1	474	53110
SPAC9E9.10c	cbh1		20	22.2	514	59865
SPCC4B3.17	cbp3		7	11	283	32972
SPBC947.14c	cbp6		1	15.5	103	12395
SPBC12D12.03	cct1		26	20.7	556	60047
SPAC1D4.04	cct2		4	8	527	56677
SPBC1A4.08c	cct3		9	15.2	528	58481
SPBC106.06	cct4		10	29.2	527	56969
SPAC1420.02c	cct5		14	15.4	546	59377
SPBC646.11	cct6		4	7.7	535	58548
SPBC337.05c	cct8		2	3.1	546	59956
SPCC1739.11c	cdc11		1	2.6	1045	118628
SPAC20G8.05c	cdc15		24	8.8	927	102119
SPAC20G8.01	cdc17		1	2.2	768	86581
SPAC1F7.05	cdc22		16	16.6	811	91999
SPAC1565.08	cdc48		3	4.9	815	90125
SPBC336.04	cdc6		1	1.1	1086	123568
SPBC21.06c	cdc7		2	2.8	1062	119291
SPBC17G9.02c	cdc73		2	5.9	371	42629
SPBC32H8.10	cdk9		7	11.2	591	68029
SPAC6G9.12	cfr1		2	2.9	620	67231
SPBC1709.08	cft1		2	1	1441	160300
SPCC24B10.12	cgi121		15	20.7	174	20050
SPAC26A3.05	chc1		16	9.5	1666	190017
SPAC23C11.11	cka1		2	7.5	332	39527
SPBP35G2.05c	cki2		4	6.7	435	49746
SPBC9B6.08	clc1		4	16.2	229	25863
SPBC2D10.17	clr1		1	1.5	1238	136677
SPBC800.03	clr3		1	2.2	687	76792
SPAC29B12.08	clr5		1	1.9	682	74637
SPBC36.05c	clr6		2	4.9	405	46112
SPACUNK12.02c	cmk1		6	3.6	335	38164
SPAC26A3.10	cnt6		2	2	923	104497
SPAC1687.12c	coq4		1	7.4	272	31100
SPBC146.12	coq6		1	3.4	466	52160
SPAC6B12.15	cpc2		7	20.7	314	34851
SPAC16C9.05	cph1		2	3.5	404	44984

SPAC19G12.10c	cpy1		1	2	1002	114237
SPAC23C4.02	crn1		1	6	601	67016
SPBC1685.08	cti6		6	6.1	424	46955
SPAC10F6.03c	cts1		14	12	600	66867
SPBP4H10.06c	cut14		1	0.8	1172	134137
SPAC56E4.04c	cut6		79	22.1	2280	256840
SPAC6F12.15c	cut9		1	2.7	671	75889
SPBC215.12	cwf10		1	3.4	983	111175
SPBC23E6.01c	cxr1		1	3.6	473	51705
SPBC29A3.18	cyt1		4	9.1	307	34340
SPAC1B1.01	deb1		4	7.7	478	51766
SPBC354.10	def1		23	14.2	963	100134
SPAC25B8.05	deg1		1	2.4	450	51604
SPCC1223.08c	dfr1		36	35.4	461	51523
SPCC1235.15	dga1		2	4.6	345	39486
SPAC1093.06c	dhc1		1	0.3	4196	484321
SPBC15C4.05	dhx29		1	2.8	1428	161459
SPBC776.02c	dis2		1	10.1	327	37636
SPAC1002.09c	dld1		4	5.7	511	54731
SPAC19G12.12	dlp1		10	18.7	294	32411
SPBC12C2.08	dnm1		2	1.8	781	87009
SPAC31G5.16c	dpm1		2	14	236	26672
SPBPJ4664.01	dps1		10	14.6	378	42047
SPAC6B12.11	drc1		1	4.5	337	38685
SPBC2F12.03c	ebs1		1	3	891	98568
SPBC428.02c	eca39		5	13.2	380	42522
SPAC1782.01	ecm29		1	2.2	1679	190967
SPAC513.01c	eft201		55	32.7	842	93231
SPBC25H2.05	egd2		53	41.6	173	18785
SPBP23A10.14c	ell1		1	5.6	533	59358
SPBC36.07	elp1		27	16.5	1253	141607
SPAC29A4.20	elp3		7	7.2	544	61850
SPAC688.11	end4		5	4.9	1092	123184
SPBC1815.01	eno101		31	27.8	439	47436
SPCC794.11c	ent3		1	1.7	476	51751
SPAC13A11.02c	erg11		1	4.8	495	56331
SPAC19A8.04	erg5		4	6.1	541	62011
SPBC646.05c	erg9		4	13.7	460	53321
SPAC1805.02c	etf2		2	6.3	254	27744
SPAC26F1.04c	etr1		2	11.8	372	41230
SPAC17A5.14	exo2		2	1.4	1328	152589
SPBC1773.01	far8		12	8	612	69494
SPAC926.09c	fas1		1701	58.9	2073	230558
SPAC4A8.11c	fas2		1675	63.6	1842	202168
SPBC19C2.07	fba1		32	45	358	39570
SPCC1235.05c	fft2		13	7.4	1284	143666
SPAC25A8.01c	fft3		10740	82.6	922	104504
SPAC1142.08	fhl1		2	2.2	743	81128
SPBC1685.13	fhn1		2	9.3	183	20273
SPBC2D10.10c	fib1		1	6.2	305	32040
SPBC1778.06c	fim1		1	4.6	614	68617

SPBC1347.02	fkbp39	2	5.5	361	39302
SPBC3E7.10	fma1	18	22.7	379	42139
SPBC14C8.03	fma2	5	14.8	426	47271
SPCC13B11.04c	fmd3	1	6.8	380	40712
SPAC1805.09c	fmt1	1	5.6	340	38041
SPAC23A1.12c	frs1	38	33.6	589	66850
SPAC3G9.06	frs2	25	22.2	499	56266
SPAC6F12.03c	fsv1	1	6.5	247	28266
SPAC1F8.06	fta5	1	10.1	385	41001
SPAC20G4.02c	fus1	1	1.6	1372	157073
SPCC1322.04	fyu1	2	5.7	506	56430
SPCC1902.01	gaf1	1	2.9	855	91777
SPBC646.12c	gap1	1	4.6	766	87540
SPAC140.02	gar2	2	4.2	500	52988
SPCC825.02	gbs1	6	6.3	506	57075
SPCC622.12c	gdh1	11	8.4	451	48790
SPAC22H10.12c	gdi1	5	4.3	440	49556
SPAC1142.06	get3	1	4.9	329	36548
SPBC25H2.16c	gga22	1	6.9	533	59482
SPAC56E4.06c	ggt2	3	10.3	611	67825
SPAC23H4.06	gln1	1	8.9	359	40018
SPAPB1E7.07	glt1	132	30	2111	232847
SPAC23H3.09c	gly1	1	5.9	376	41318
SPBC354.12	gpd3	170	41.2	335	35675
SPAC26F1.06	gpm1	4	11.8	211	23765
SPBPJ4664.06	gpt1	89	29.4	1448	165468
SPBC26H8.08c	grn1	1	3.6	470	52434
SPBC26H8.06	grx4	2	15.6	244	27099
SPAC688.04c	gst3	1	10.2	225	26041
SPBC354.01	gtp1	2	4.1	363	40749
SPCC777.05	gtr2	1	6.7	314	35592
SPBC2F12.14c	gua1	9	8.2	524	57026
SPAC17A5.15c	gus1	39	25.3	716	80750
SPAC29A4.16	hal4	23	24.8	636	69115
SPAC2F3.09	hem1	12	17.2	558	60960
SPCC320.09	hem15	1	5.5	384	42714
SPAC1834.03c	hhf1	2	11.7	103	11423
SPBC3H7.15	hhp1	2	3.8	365	42450
SPAC1834.04	hht1	6	30.1	136	15357
SPBC31F10.13c	hip1	11	5.7	932	103686
SPBC31F10.14c	hip3	3	4.2	1630	186700
SPAC25G10.05c	his1	6	7.4	310	34029
SPBC1711.13	his2	2	8.9	439	47554
SPBC2G5.06c	hmt2	27	37.3	459	51575
SPBC725.09c	hob3	2	5.3	264	30094
SPAC2E12.02	hsf1	4	5.9	609	66948
SPBC16D10.08c	hsp104	3	3.5	905	100507
SPBC3E7.02c	hsp16	1	4.9	143	15968
SPAC926.04c	hsp90	16	11.8	704	80596
SPAC3H1.11	hsr1	9	7.9	582	64126
SPCC132.02	hst2	1	4.8	332	37916

SPCC622.08c	hta1	19	28	132	13878
SPAC19G12.06c	hta2	19	28.2	131	13776
SPCC622.09	htb1	89	63.5	126	13819
SPAC4F8.07c	hxk2	2	6.8	455	50920
SPBC902.05c	idh2	1	3.4	378	41060
SPAC6G10.08	idp1	1	3.6	418	47293
SPAC144.02	iec1	5	13.7	249	28348
SPAC23G3.04	ies4	6	21.1	194	21142
SPAC222.04c	ies6	1	12.8	117	13476
SPBP35G2.07	ilv1	62	32.6	669	73194
SPBC56F2.12	ilv5	5	17.6	404	45189
SPCC737.03c	ima1	1	4.4	615	70434
SPBC11C11.02	imp2	1	2.8	670	75167
SPAC29B12.01	ino80	17	8.2	1604	183048
SPBC646.09c	int6	13	13.8	501	57114
SPAC23C11.05	ipp1	1	5.5	289	32468
SPBC8D2.06	irs1	2	4.4	1064	122915
SPBC365.12c	ish1	28	20.2	684	75699
SPAC19G12.14	its3	7	8.4	742	83748
SPBC19G7.16	iws1	7	11.4	428	49115
SPBC14F5.03c	kap123	14	14.1	1067	117834
SPAC1B1.03c	kap95	1	2.8	863	94747
SPBC1718.03	ker1	1	25.9	147	16976
SPBC1271.12	kes1	11	24	388	44592
SPBC4F6.06	kin1	3	4.6	891	98749
SPAC1834.07	klp3	1	5.2	554	61939
SPBC17G9.03c	krs1	1	1.4	591	67494
SPCC794.07	lat1	46	23.4	483	52061
SPBC18H10.02	lcf1	2	5.6	676	75966
SPBP4H10.11c	lcf2	1	2.6	689	75791
SPBC13E7.08c	leo1	2	4.4	429	48733
SPBC1A4.02c	leu1	16	22.4	371	39733
SPAC9E9.03	leu2	9	8.8	758	82783
SPBC3E7.16c	leu3	1	2.9	584	64042
SPAC22F3.06c	lon1	3	2.7	1067	118642
SPAPJ696.02	lsb4	1	3.7	430	46374
SPBC31F10.07	lsb5	1	3.3	304	34195
SPAC23E2.02	lsd2	1	1.3	1273	142490
SPAP7G5.04c	lys1	3	2.7	1419	156894
SPAC31G5.04	lys12	1	2.5	362	39256
SPAC227.18	lys3	10	9.2	368	41393
SPBC1105.02c	lys4	9	21.3	418	46293
SPCC794.12c	mae2	2	4.6	565	62535
SPBC1734.11	mas5	43	36.6	407	44821
SPCC4G3.14	mdj1	10	15.2	528	57189
SPBC146.01	med15	3	3	1063	118463
SPBC28F2.02	mep33	2	7.5	292	33282
SPAC13G7.06	met16	6	14.7	266	30561
SPAC9.09	met26	2	3.7	764	85340
SPCC1259.14c	meu27	1	5	496	56506
SPCC24B10.11c	mft1	1	13.4	202	23282

SPAC26H5.05	mga2	1	1.1	1151	127698
SPBC25B2.07c	mmb1	3	3	501	53763
SPAC30C2.02	mmd1	1	6.9	318	35689
SPCC736.12c	mmi1	20	18.9	488	54539
SPAC637.07	moe1	17	27	567	62637
SPBC1826.01c	mot1	2	2.3	1953	217630
SPBP8B7.15c	mpe1	1	4.1	482	53163
SPCC1906.01	mpg1	26	36.9	363	39719
SPBC13G1.02	mpg2	9	20	414	46096
SPBC19G7.01c	msh2	1	3.4	982	109739
SPCC285.16c	msh6	1	1	1254	141511
SPAC2C4.17c	msy2	1	3	840	94644
SPCC417.07c	mto1	2	2.2	1115	128469
SPBP19A11.03c	mts4	37	24.6	891	97999
SPAC1F3.09	mug161	1	5.5	561	63217
SPAC6B12.08	mug185	1	3.2	380	45131
SPBC14F5.11c	mug186	3	6	586	67002
SPAC56F8.05c	mug64	3	7	286	31748
SPCC1902.02	mug72	49	38.2	574	62847
SPAC10F6.07c	mug94	1	9.6	188	21532
SPAC3H8.09c	nab3	1	2.3	738	82430
SPCC364.06	nap1	30	39.9	393	44348
SPBC2D10.11c	nap2	1	7.7	379	43371
SPAC20G8.09c	nat10	2	4.1	1033	116463
SPAC637.08	nbp35	4	6.6	317	34261
SPBC16A3.15c	nda2	5	12.1	455	51152
SPBC26H8.07c	nda3	16	34.4	448	49472
SPBC947.15c	nde1	2	4.9	551	60781
SPAC3A11.07	nde2	56	37.7	551	61675
SPBC12D12.08c	ned8	1	32.1	78	8661
SPBC21D10.11c	nfs1	3	6.6	498	55197
SPBC28F2.10c	ngg1	1	3.3	551	62407
SPAC1782.10c	nhp2	8	31.8	154	17178
SPCC70.05c	nnk1	6	6.4	781	85910
SPCC4G3.15c	not2	4	20.5	176	20596
SPAC1B3.05	not3	1	3.8	630	71715
SPAC2F7.11	nrd1	5	18.9	529	57761
SPBC1773.10c	nrs1	4	15.5	568	63870
SPBC660.07	ntp1	2	5.4	735	84603
SPBC4C3.05c	nuc1	15	7.8	1689	189244
SPCC162.08c	nup211	13	6.8	1837	211458
SPAC4F10.18	nup37	1	9	391	42776
SPBC13A2.02	nup82	2	4.5	803	90612
SPAC3C7.14c	obr1	1	8.4	202	21899
SPCC1020.10	oca2	8	7.2	650	73232
SPCC16A11.10c	oca8	1	14	129	14252
SPBC16A3.08c	oga1	2	3.5	284	30949
SPBC19C7.12c	omh1	2	9.7	390	46514
SPAC27D7.04	omt2	6	16.7	96	11101
SPBC685.09	orc2	1	4.1	535	61023
SPAC23H4.01c	osh3	4	2.9	945	106810

SPAP8A3.09c	paa1	7	13.4	590	66617
SPAC227.07c	pab1	1	2.8	463	52795
SPAC57A7.04c	pabp	22	17	653	71513
SPAC25G10.09c	pan1	12	3.6	1794	193277
SPBC32F12.06	pch1	6	16.1	342	38286
SPAC17G8.14c	pck1	17	16.2	988	111784
SPAC6G9.06c	pcp1	1	1.5	1208	140763
SPAC21E11.03c	pcr1	7	8.8	171	19348
SPAC3H1.10	pcs2	3	8	414	46741
SPAC26F1.03	pda1	6	19.3	409	45138
SPBC30D10.13c	pdb1	5	12.8	366	39624
SPBC3D6.13c	pdi3	3	5.8	726	81237
SPCC1259.09c	pdx1	1	3.3	456	50884
SPCC553.03	pex1	2	6.3	937	105905
SPBC582.09	pex11	5	10.1	238	26479
SPBC16H5.02	pfk1	46	21.4	942	102555
SPBC14F5.04c	pgk1	19	17.6	414	43964
SPCC4G3.07c	phf1	3	12.8	461	51285
SPBP4G3.02	pho1	5	13.2	453	50557
SPBC725.11c	php2	5	2.4	334	34893
SPBC3B8.02	php5	2	3.1	415	46674
SPCC736.15	pil1	1	10.3	351	39806
SPBC725.08	pir2	1	4.8	609	70865
SPBC106.10	pka1	1	2	512	57578
SPAC1A6.04c	plb1	3	4.9	613	67119
SPAC2F7.16c	pld1	1	1	1369	157730
SPBC106.11c	plg7	17	39	438	49847
SPAC1071.10c	pma1	31	12.9	919	99884
SPBC1289.04c	pob1	5	2.9	871	93773
SPBC14C8.14c	pol5	1	1.7	959	109719
SPBC1718.01	pop1	2	1.3	775	87817
SPAC823.15	ppa1	1	8.4	309	35291
SPBC16H5.07c	ppa2	10	11.5	322	36489
SPBP4H10.04	ppb1	13	18.4	554	63714
SPCC4B3.18	ppc1	1	7.3	316	36242
SPBC336.14c	ppk26	1	6.8	589	67268
SPBC6B1.02	ppk30	1	1.5	953	105897
SPBC725.06c	ppk31	1	1.3	1032	120269
SPBP23A10.10	ppk32	22	19.6	749	82824
SPBC19F5.05c	ppp1	4	4.1	607	69189
SPCC1442.06	pre8	1	6.9	245	26404
SPAC821.11	pro1	4	4	451	48707
SPAC27F1.09c	prp10	1	1.5	1188	135151
SPBC146.07	prp2	1	6.2	517	58933
SPBC19C7.06	prs1	1	2.8	716	78875
SPCC830.07c	psi1	5	13.2	379	40260
SPAC1006.01	psp3	9	14	451	48733
SPAC110.04c	pss1	3	5.3	720	80375
SPBC12C2.10c	pst1	4	3.8	1522	171455
SPBC1734.16c	pst3	4	6.7	1154	132881
SPCC4F11.02	ptc1	3	8.4	347	38676

SPAC4A8.03c	ptc4		1	5	383	43569
SPAC19D5.04	ptr1		2	1.1	3227	365036
SPAC13F5.02c	ptr6		4	11.5	393	45000
SPCP1E11.11	puf6		15	15.3	642	72635
SPCC63.12c	pup3		1	9.8	204	22661
SPCC126.03	pus1		3	8.4	534	60324
SPAC22F8.02c	pvg5		1	7.8	372	43087
SPBC1711.16	pwp1		2	6.2	516	57337
SPAC4H3.10c	pyk1		24	34.8	509	55515
SPBC17G9.11c	pyr1		1	0.9	1185	130861
SPAC57A7.08	pzh1		2	3.5	515	57131
SPCC553.02	qns1		1	2.4	700	79520
SPBC342.02	qrs1		1	1.7	811	92073
SPAC8E11.02c	rad24		12	26.3	270	30082
SPAC17A2.13c	rad25		2	17.8	270	30369
SPAC1556.01c	rad50		2	4	1285	149028
SPBC17A3.04c	rar1		31	25.2	782	88881
SPBC23E6.07c	rfc1		1	1.3	934	103489
SPBC83.14c	rfc5		3	9.2	358	40403
SPBC3F6.05	rga1		1	1.6	1150	130602
SPAC1F7.04	rho1		8	11.9	202	22523
SPAC20H4.11c	rho5		8	12	200	22408
SPBC1734.06	rhp18		1	3.1	387	43438
SPBC1709.12	rid1		4	9.8	367	42550
SPBC1703.05	rio2		4	4.2	336	38956
SPBC16H5.06	rip1		1	3.9	228	24740
SPBC14F5.06	rli1		5	6.1	593	66590
SPBC902.04	rmn1		8	12.2	589	66899
SPCC757.09c	rnc1		2	6.8	398	43377
SPBP23A10.07	rpa2		14	13.5	1174	131687
SPAC2F3.03c	rpa49		4	15.5	425	48066
SPBC28F2.12	rpb1		3	2.1	1752	194161
SPAC1B3.12c	rpb10		1	35.2	71	8276
SPAC23G3.01	rpb2		3	3.6	1210	137849
SPAC23C4.15	rpb5		3	10.5	210	23915
SPCC330.13	rpc37		1	5.4	242	27385
SPBC1289.07c	rpc40		5	15.5	348	39155
SPAC637.10c	rpn10		1	11.1	243	27140
SPAC31G5.13	rpn11		2	14.3	308	34572
SPBC16G5.01	rpn12		4	18.5	270	30899
SPBC342.04	rpn1301		6	32.3	291	31821
SPCC16A11.16c	rpn1302		4	12.6	388	43903
SPBC17D11.07c	rpn2		7	6.5	965	107276
SPBC119.01	rpn3		14	9.7	497	57346
SPAPB8E5.02c	rpn502		13	12.4	443	51618
SPAC23G3.11	rpn6		9	7.1	421	47341
SPBC582.07c	rpn7		19	14.2	409	46680
SPCC1682.10	rpn8		2	7.4	324	35595
SPAC607.05	rpn9		3	16.3	381	43457
SPBC16C6.07c	rpt1		7	11.9	438	48965
SPBC4.07c	rpt2		17	18.5	448	50060

SPCC576.10c	rpt3		22	34.7	389	43553
SPCC1682.16	rpt4		6	16.5	388	43608
SPAC3A11.12c	rpt5		20	21.2	438	48836
SPBC23G7.12c	rpt6		5	4.7	403	45069
SPAC1F3.01	rrp6		12	4.4	777	89560
SPAC1F3.07c	rsc58		2	10.2	403	44621
SPBC1703.02	rsc9		5	5.1	780	87995
SPAC6F12.02	rst2		6	16.9	567	62560
SPAC22F8.07c	rtf1		1	3.9	466	55219
SPAPB8E5.09	rvb1		15	25.7	456	50053
SPBC83.08	rvb2		13	26.9	465	51562
SPCC1840.03	sal3		4	6.8	1095	121869
SPBC14F5.05c	sam1		16	24.6	382	41832
SPCC1672.02c	sap1		3	6.7	254	29127
SPAC22A12.09c	sap114		4	11.9	481	54409
SPBC21C3.05	sap62		4	18.4	217	24987
SPBC31F10.06c	sar1		3	21.6	190	21280
SPBC18H10.04c	sce3		9	20.6	388	42514
SPBC646.16	scl1		1	7.4	244	26539
SPBC119.06	sco1		2	6.1	263	30240
SPBC1D7.02c	scr1		2	3.4	565	59713
SPAC17C9.12	scs22		2	9.4	319	34005
SPCC16C4.07	scw1		9	17.3	561	60394
SPBC106.14c	sda1		1	4	719	81251
SPCC18.11c	sdc1		2	21.1	109	11856
SPAC1556.02c	sdh1		4	5	641	70466
SPCC584.05	sec1		7	6.5	693	79638
SPBC215.15	sec13		2	11.1	297	32568
SPAC29B12.07	sec16		2	0.6	1995	218089
SPAC1834.11c	sec18		40	24.6	792	87554
SPAC23C4.10	sec2		5	4.2	527	59053
SPAC57A7.10c	sec21		7	8.8	905	100936
SPCC31H12.07	sec231		1	4	759	84845
SPBC146.14c	sec26		3	3.8	940	104700
SPBC8D2.20c	sec31		1	2.5	1224	132499
SPCC126.15c	sec65		7	17.1	199	21955
SPAC29B12.02c	set2		1	3.3	798	90679
SPCC16A11.14	sfh1		5	6.5	418	46839
SPAC7D4.02c	sfp47		1	2.4	415	46782
SPAC1002.10c	sgt1		2	4.2	590	68157
SPBC1604.14c	shk1		4	1.8	658	72358
SPAC1F5.09c	shk2		1	3.7	589	66766
SPAC18G6.04c	shm2		3	5.1	472	52081
SPAC10F6.01c	sir1		10	8.2	1473	163844
SPBC16D10.07c	sir2		5	8.2	475	53443
SPBC1709.05	sks2		229	53.7	613	67206
SPAC57A10.10c	sla1		2	4.7	298	34616
SPAC1851.02	slc1		3	12.2	279	31393
SPAC821.03c	slf1		2	5.2	485	53991
SPAC637.13c	slm1		3	5	498	56088
SPBC15D4.03	slm9		5	6.1	807	90433

SPCC5E4.06	smc6	1	1.5	1140	130929
SPBC30D10.17c	smi1	2	5.6	504	55558
SPAC1250.01	snf21	2	2.2	1199	140089
SPAC607.03c	snu13	1	23.2	125	13535
SPCC594.05c	spf1	3	3.3	424	48683
SPBC1289.03c	spi1	1	4.2	216	24555
SPBC19F8.01c	spn7	1	6.3	428	49298
SPAC1F3.06c	spo15	1	0.7	1957	222785
SPAC3H8.10	spo20	1	6.3	286	32746
SPAC4F8.12c	spp42	4	1.9	2363	274554
SPBP8B7.19	spt16	4	7.9	1019	116451
SPBC21C3.16c	spt4	10	48.6	105	11878
SPAC23C4.19	spt5	17	7.6	990	109014
SPBC25H2.11c	spt7	11	11.8	979	111504
SPBC14C8.17c	spt8	2	2.9	526	58418
SPBC354.05c	sre2	4	3	793	86834
SPCC1322.08	srk1	2	6.4	580	66136
SPBC11C11.08	srp1	2	5.1	275	31069
SPAC19B12.09	srp14	4	11.3	106	11718
SPAC16.02c	srp2	14	8.5	365	42566
SPCC320.10	srp72	1	3.6	561	63281
SPCC1739.13	ssa2	116	46.4	647	70233
SPBC23E6.09	ssn6	4	3.4	1102	121516
SPCC297.03	ssp1	2	5.5	652	73992
SPCC74.03c	ssp2	1	2.3	576	65996
SPAC17G6.10	ssr1	5	11.8	527	59639
SPAC23H3.10	ssr2	1	4.8	503	57685
SPAC23G3.10c	ssr3	1	5.4	425	49759
SPAC3G9.04	ssu72	1	8.6	197	22596
SPBC776.09	ste13	1	2.9	485	54795
SPBC12C2.02c	ste20	1	2.1	1309	147412
SPAC1071.12c	stp1	44	42.3	156	17391
SPCC16C4.09	sts5	32	17.8	1066	117603
SPBC577.06c	stt4	3	2.3	1877	213961
SPAC24B11.06c	sty1	2	2.9	349	40222
SPAC4D7.05	sum1	4	12.8	328	36811
SPCC1795.11	sum3	24	29.9	636	69759
SPCC584.04	sup35	12	10	662	72556
SPAC9G1.13c	swc4	5	17.8	437	50384
SPAC11E3.01c	swr1	3	1.9	1288	149456
SPBC2G2.02	syj1	37	22.9	1076	121822
SPBC577.13	syj2	1	2.2	889	101708
SPBC4C3.06	syp1	7	11.5	818	90757
SPBC21H7.02	taf10	4	20.9	215	23526
SPAC2G11.14	taf111	17	15.3	979	111014
SPAC3A12.05c	taf2	35	24	1172	133909
SPCC16C4.18c	taf6	4	3.8	452	50273
SPBC15D4.14	taf73	2	4.8	642	72251
SPAC12G12.05c	taf9	1	10.4	163	18448
SPBC19G7.13	tbf1	1	3.7	485	54604
SPBC660.11	tcg1	12	19.8	348	37872

SPBC32F12.11	tdh1		920	64.9	336	35870
SPCC794.09c	tef101		3187	75.2	460	49661
SPAC23A1.10	tef102		2909	75.2	460	49675
SPCC417.08	tef3		55	24.6	1047	115802
SPCC1672.08c	tfa2		6	12.6	285	32162
SPCC1620.09c	tfg1		2	5.6	539	61449
SPAC20H4.03c	tfs1		17	19.8	293	32734
SPBC839.16	thf1		1	1.7	937	101202
SPAC1D4.14	tho2		1	1.4	1628	188832
SPCC965.05c	thp1		1	4.3	325	36574
SPAC3G9.09c	tif211		13	29.4	306	34526
SPAC32A11.04c	tif212		22	18.7	321	35961
SPBC17G9.09	tif213		37	37	446	48781
SPAC4D7.09	tif223		4	3.6	468	51632
SPAC21E11.06	tif224		3	10.5	467	51583
SPAC8C9.15c	tif225		19	17.6	678	76326
SPBC17D11.05	tif32		33	15.5	932	107071
SPAC4A8.16c	tif33		34	25.2	918	104367
SPBC18H10.03	tif35		7	24.1	282	31494
SPAC16E8.15	tif45		1	9.6	218	24958
SPAC17C9.03	tif471		38	14.3	1403	154034
SPAC26H5.10c	tif51		9	42.7	157	17152
SPBC106.05c	tim11		3	33.7	92	10711
SPAC824.06	tim14		1	12.9	140	15271
SPBC713.10	tim16		4	21.1	128	14120
SPCC16A11.09c	tim23		1	11	210	22622
SPBC14C8.02	tim44		7	7.7	427	48550
SPBC1347.04	tim54		2	5.8	347	39803
SPAC1399.05c	toe1		1	4.9	529	60558
SPAC27D7.14c	tpr1		1	2	1039	119151
SPCC576.03c	tpx1		1	9.9	192	21191
SPBP16F5.03c	tra1		1	0.4	3699	422341
SPBC25D12.05	trm1		1	5.3	548	60297
SPAC17D4.04	trm401		1	2	654	74555
SPBC1539.09c	trp1		8	10	759	83040
SPAC19A8.15	trp2		23	17.5	697	75437
SPCC1442.09	trp3		1	2.9	489	54960
SPBC3F6.03	trr1		1	5.9	322	34618
SPBC25H2.02	trs1		22	12.2	703	80138
SPCC285.14	trs130		1	2.7	1150	132875
SPBC577.08c	txl1		1	6.2	290	31889
SPAC17G6.14c	uap56		4	10.1	434	49231
SPCC777.10c	ubc12		3	11.3	177	20062
SPBC119.02	ubc4		2	18.4	147	16476
SPBC211.07c	ubc8		2	8.2	184	20990
SPCC1494.05c	ubp12		1	2.5	979	111967
SPCC1682.12c	ubp16		4	8.3	457	51582
SPAC328.06	ubp2		2	1.8	1141	130297
SPBP8B7.21	ubp3		18	24	512	58082
SPBC1703.12	ubp9		1	3.8	585	66789
SPBC21C3.11	ubx4		4	4.9	425	46697

SPBC21D10.05c	ucp3	6		7.2	601	65542
SPAC16C9.06c	upf1	1		1.9	925	104529
SPAC22G7.06c	ura1	202		32	2244	248306
SPAC57A10.12c	ura3	4		3.2	443	48296
SPBC725.15	ura5	1		7	215	23716
SPAC4D7.13	usp104	2		6	695	81931
SPBC839.10	usp107	3		6.3	695	81044
SPBC428.19c	utp15	6		5.9	494	54112
SPCC1827.01c	utp25	4		5.7	652	75652
SPCC550.14	vgl1	11		9.3	1279	141231
SPAC343.05	vma1	1		4.2	619	68806
SPAC637.05c	vma2	5		8.3	503	55841
SPAC767.01c	vps1	1		1.9	678	75791
SPBC16C6.06	vps10	1		2.5	1466	165061
SPBC16C6.02c	vps1302	5		1.2	3004	339017
SPAC1142.07c	vps32	1		10.8	222	25302
SPAC2G11.06	vps4	1		6.9	432	48402
SPAC17A2.06c	vps8	1		2.7	1272	146210
SPAC144.18	vrg4	1		6.7	345	38019
SPBC13E7.09	vrp1	3		6.1	309	31225
SPBC1709.02c	vrs1	3		4.7	980	111317
SPCC1322.14c	vtc4	14		15.1	721	83635
SPBC409.07c	wis1	1		1.8	605	64762
SPAC9G1.02	wis4	1		0.8	1401	160537
SPAC4F10.15c	wsp1	6		11.3	574	59606
SPAC19A8.03	ymr1	11		14.1	559	64153
SPCC1672.05c	yrs1	5		3.7	401	44576
SPAC31F12.01	zds1	1		2	938	103098
SPBC1778.01c	zuo1	11		10.6	442	50209
SPBP4H10.15	Unassigned	122		35.7	912	99223
SPBC2F12.05c	Unassigned	93		29	1310	148475
SPAC17H9.12c	Unassigned	87		69.9	266	29375
SPBC646.08c	Unassigned	75		43.2	516	58692
SPAC24C9.12c	Unassigned	73		50.7	467	51861
SPAC1F8.07c	Unassigned	50		38.8	569	61634
SPAC4H3.01	Unassigned	45		16.3	392	44769
SPCC584.01c	Unassigned	40		20.3	1006	111353
SPCC23B6.01c	Unassigned	38		29.4	489	55857
SPAC30C2.04	Unassigned	28		20.2	450	50606
SPAC3A11.10c	Unassigned	25		23.5	409	46048
SPAC2F7.05c	Unassigned	24		37.5	395	44945
SPAC25G10.08	Unassigned	24		17.2	725	84035
SPAC31A2.12	Unassigned	21		25	596	65902
SPAC18G6.12c	Unassigned	21		36.9	309	35297
SPBPB7E8.02	Unassigned	20		20.3	749	82211
SPCC330.03c	Unassigned	18		41.4	145	16337
SPAC1B1.02c	Unassigned	17		21.6	537	60708
SPBC25B2.10	Unassigned	17		19.5	307	34082
SPAC17A5.13	Unassigned	16		30.2	235	26493
SPBC83.16c	Unassigned	15		9.9	563	63485
SPBC4C3.07	Unassigned	15		17.9	302	33251

SPBC29B5.04c	Unassigned	15	24.1	605	68509
SPAC1006.07	Unassigned	14	12.8	392	44436
SPAC694.02	Unassigned	13	4.4	1717	195057
SPBC3B8.03	Unassigned	13	27.6	450	49938
SPCC1827.02c	Unassigned	12	16.9	362	41751
SPAC17H9.13c	Unassigned	12	22.4	402	43934
SPAC29A4.02c	Unassigned	12	24.4	409	45701
SPCC11E10.07c	Unassigned	11	17.6	341	37773
SPBC1709.14	Unassigned	11	27.3	333	39241
SPBC2D10.04	Unassigned	10	10.6	658	72732
SPAC1751.03	Unassigned	10	8	402	45085
SPAC167.05	Unassigned	10	12	601	65948
SPBC365.09c	Unassigned	10	15.1	304	35453
SPCC1672.07	Unassigned	10	9.5	902	100569
SPBC26H8.11c	Unassigned	8	18.3	175	19591
SPAC29E6.06c	Unassigned	8	10.6	754	85555
SPBC19F5.04	Unassigned	8	21.4	519	56813
SPCC14G10.04	Unassigned	8	16.9	497	52625
SPAC8E11.10	Unassigned	8	52.5	255	27437
SPBC13E7.03c	Unassigned	7	9.8	713	77869
SPAC6F6.12	Unassigned	7	24.9	401	46376
SPCC594.01	Unassigned	7	13.1	791	88749
SPAC823.08c	Unassigned	7	16.8	465	51864
SPAC821.05	Unassigned	7	22.1	357	39781
SPAC926.08c	Unassigned	6	10.4	317	36071
SPCC1739.01	Unassigned	6	5.9	547	57978
SPAC56E4.03	Unassigned	6	13.7	474	53167
SPBC19G7.10c	Unassigned	6	10.3	754	84117
SPBC17D1.05	Unassigned	5	13.9	368	37873
SPBC24C6.04	Unassigned	5	4.2	548	60219
SPAC25G10.01	Unassigned	5	4.7	297	33564
SPAC23A1.17	Unassigned	5	3.7	1611	170527
SPAC24B11.07c	Unassigned	5	8.9	561	62464
SPBC26H8.04c	Unassigned	5	3.3	1496	169994
SPCC23B6.04c	Unassigned	5	11.6	1008	110725
SPCC63.14	Unassigned	5	9.1	1184	131057
SPAC458.02c	Unassigned	4	4.7	468	53736
SPBC660.16	Unassigned	4	13.2	492	53680
SPCC1281.06c	Unassigned	4	11.3	479	54438
SPBC21C3.15c	Unassigned	4	8.4	522	57571
SPAC18G6.05c	Unassigned	4	1.9	2670	297336
SPCC4G3.13c	Unassigned	3	19.1	215	23524
SPAC1952.04c	Unassigned	3	10.1	158	18209
SPAC2G11.10c	Unassigned	3	5.2	401	44416
SPBC1711.08	Unassigned	3	3.9	336	37420
SPBC646.10c	Unassigned	3	3.8	497	55388
SPBC1711.07	Unassigned	3	3.5	480	53834
SPAC29A4.15	Unassigned	3	3.3	450	51643
SPAC17H9.04c	Unassigned	3	2.6	604	66396
SPBC29A3.09c	Unassigned	3	2	736	83762
SPAC1093.03	Unassigned	3	1.9	828	95613

SPBC8D2.18c	Unassigned	3	12.5	433	47383
SPAP8A3.07c	Unassigned	3	11	372	40638
SPBC25B2.03	Unassigned	3	10.8	554	61805
SPAC12G12.07c	Unassigned	3	10.2	412	45724
SPBC428.15	Unassigned	3	9.8	409	44462
SPAC343.14c	Unassigned	3	8.7	393	43221
SPBC2G5.05	Unassigned	3	8.2	685	75182
SPAC6G9.15c	Unassigned	3	8.2	498	55323
SPAC17G8.06c	Unassigned	3	6.5	598	63927
SPAC1687.09	Unassigned	3	3.9	1379	150017
SPBC16E9.10c	Unassigned	3	3.9	779	86179
SPCC70.06	Unassigned	3	3.5	458	52334
SPBC12C2.11	Unassigned	3	3.4	696	77322
SPCC1183.07	Unassigned	3	2.2	1690	187523
SPCC24B10.04	Unassigned	3	31.7	199	23366
SPBC1703.07	Unassigned	3	8	615	67204
SPBP35G2.14	Unassigned	3	7.3	1060	113647
SPBC83.17	Unassigned	2	9.5	148	15979
SPBC577.04	Unassigned	2	7	200	23497
SPAC3A12.13c	Unassigned	2	6.6	274	30532
SPAPB1A10.13	Unassigned	2	6.4	529	56250
SPBC12C2.04	Unassigned	2	6.2	384	43032
SPAPB24D3.08c	Unassigned	2	6	349	38613
SPBC36B7.08c	Unassigned	2	5.7	244	28637
SPBC1604.09c	Unassigned	2	4.6	260	29602
SPBC17D11.08	Unassigned	2	4.1	435	47743
SPAC23G3.06	Unassigned	2	3.7	508	55759
SPBC3E7.11c	Unassigned	2	3.7	355	40531
SPBC4F6.13c	Unassigned	2	3.3	736	82526
SPAC926.06c	Unassigned	2	3.1	621	69485
SPAC23C4.05c	Unassigned	2	2.8	431	49532
SPAC4A8.06c	Unassigned	2	2.6	578	66110
SPCC1235.01	Unassigned	2	2.1	658	69696
SPACUNK4.16c	Unassigned	2	1.7	944	106818
SPBC1685.14c	Unassigned	2	1.6	801	92427
SPBC31F10.16	Unassigned	2	1.6	679	77585
SPAC11D3.16c	Unassigned	2	26	131	14846
SPAC1527.03	Unassigned	2	11.6	475	53233
SPAC22A12.16	Unassigned	2	10	492	53943
SPAC144.12	Unassigned	2	9.9	274	29329
SPAC683.02c	Unassigned	2	4.6	218	24644
SPAC56F8.03	Unassigned	2	3.8	1079	119931
SPBC354.08c	Unassigned	2	3.6	865	99207
SPBC16H5.08c	Unassigned	2	3.2	618	69213
SPBC3H7.03c	Unassigned	2	3	1009	114164
SPCC550.11	Unassigned	2	2.7	1029	116467
SPBC29A10.16c	Unassigned	1	22.6	124	13910
SPAC29B12.11c	Unassigned	1	14.4	174	19704
SPCC162.06c	Unassigned	1	10.5	210	23591
SPCC126.13c	Unassigned	1	10.3	145	16849
SPCC16C4.16c	Unassigned	1	9.7	206	23854

SPBC21C3.17c	Unassigned	1	8.6	186	21295
SPCC1183.02	Unassigned	1	8.2	220	25504
SPBC1271.07c	Unassigned	1	7.4	163	18479
SPBC18E5.01	Unassigned	1	7	342	38221
SPAC1952.09c	Unassigned	1	6.7	521	57916
SPAC607.08c	Unassigned	1	5.9	579	63631
SPAC4F10.06	Unassigned	1	5.9	388	44884
SPCC1827.03c	Unassigned	1	5.5	512	56274
SPCC1223.01	Unassigned	1	5.3	732	82699
SPAC26F1.07	Unassigned	1	5.3	321	36192
SPAC1071.11	Unassigned	1	5.3	244	27043
SPBC713.09	Unassigned	1	5.1	395	44770
SPBC336.02	Unassigned	1	4.9	307	34678
SPBC1677.03c	Unassigned	1	4.7	600	66544
SPAC13F5.05	Unassigned	1	4.7	363	40764
SPAC1142.04	Unassigned	1	4.4	707	81128
SPCC320.05	Unassigned	1	4.3	667	73468
SPAC977.14c	Unassigned	1	4.3	351	39915
SPBC4.02c	Unassigned	1	4.1	437	50010
SPBC9B6.11c	Unassigned	1	4	502	57651
SPBC336.05c	Unassigned	1	4	378	43827
SPCC1620.06c	Unassigned	1	4	321	34779
SPAC3H8.08c	Unassigned	1	3.9	563	64691
SPBP18G5.02	Unassigned	1	3.9	534	61429
SPAC3H8.02	Unassigned	1	3.8	444	51474
SPAC8C9.04	Unassigned	1	3.7	647	67036
SPAC732.02c	Unassigned	1	3.7	408	46948
SPAC12G12.09	Unassigned	1	3.6	977	109713
SPCC1739.06c	Unassigned	1	3.4	496	54712
SPAC14C4.11	Unassigned	1	3.3	734	85445
SPAC25B8.16	Unassigned	1	3.2	698	79931
SPCC584.15c	Unassigned	1	3.2	594	65327
SPAC222.13c	Unassigned	1	3	592	68640
SPBC428.10	Unassigned	1	2.9	751	82579
SPBC1347.05c	Unassigned	1	2.9	381	42384
SPAC57A7.12	Unassigned	1	2.8	566	61286
SPCC962.01	Unassigned	1	2.4	1429	156031
SPAC6G10.09	Unassigned	1	2.4	808	92890
SPAC222.14c	Unassigned	1	2.4	762	87510
SPCC4B3.03c	Unassigned	1	2.2	679	74306
SPBP4H10.07	Unassigned	1	1.7	583	64385
SPBPJ4664.04	Unassigned	1	1.4	1207	136369
SPCC970.08	Unassigned	1	0.9	967	108719

Supplementary Table 2. Strains used in this study

strain		Genotype	source
CBL13	h-	ade6-M210 leu1-32 ura4-D18	This study
CBL14	h+	ade6-M210 leu1-32 ura4-D18	This study
CBL149	h+	ade6-M210 leu1-32	This study
CBL1857	h+	fft1Δ::kanMX6 ade6-M210 leu1-32	This study
CBL1950	h+	fft2Δ::natMX6 ade6-M210 leu1-32	This study
CBL1860	h+	fft3Δ::natMX6 ade6-M210 leu1-32	This study
CBL1953	h+	fft1Δ::kanMX6 fft2Δ::natMX6 ade6-M210 leu1-32	This study
CBL1957	h+	fft1Δ::hphMX6 fft3Δ::natMX6 ade6-M210 leu1-32	This study
CBL2161	h+	fft2Δ::natMX6 fft3Δ::kanMX6 ade6-M210 leu1-32	This study
CBL3653	h+	fft2Δ::natMX6 fft3Δ::kanMX6 ade6-M210 leu1-32	This study
CBL1969	h+	fft1Δ::kanMX6 fft2Δ::natMX6 fft3Δ::hphMX6 ade6-M210 leu1-32	This study
CBL3306	h-	fft3Δ::hphMX6 ade6-M210 leu1-32 ura4-D18	This study
CBL3307	h+	fft3Δ::hphMX6 ade6-M210 leu1-32 ura4-D18	This study
CBL3308	h+	fft3Δ::hphMX6 ade6-M210 leu1-32	This study
CBL27	h+	hrp1Δ::kanMX6 ade6-M210 leu1-32 ura4-D18	Shim *et al* . 2012
CBL31	h+	hrp3Δ::kanMX6 ade6-M210 leu1-32 ura4-D18	Shim *et al* . 2012
CBL1296	h-	ies6Δ::natMX6 ade6-M210 leu1-32 ura4-D18	This study
CBL3443	h-	snf21-36ts leu1-32 (HU2262)	Yamada *et al* . 2008
CBL1253	h-	snf22Δ::kanMX6 ade6-M210 leu1-32 ura4-D18	This study
CBL1247	h-	swr1Δ::kanMX6 ade6-M210 leu1-32 ura4-D18	This study
CBL2513	h-	spt16-18-kan ade6-M210 leu1-32 ura4-D18	Choi *et al* . 2012
CBL4050	h+	fft2-5xFLAG-hphMX6 ade6-M210 leu1-32 ura4-D18	This study
CBL4051	h+	fft3-5xFLAG-hphMX6 ade6-M210 leu1-32 ura4-D18	This study
CBL4052	h+	ino80-5xFLAG-hphMX6 ade6-M210 leu1-32 ura4-D18	This study
CBL4053	h+	snf21-5xFLAG-hphMX6 ade6-M210 leu1-32 ura4-D18	This study
CBL4054	h+	snf22-5xFLAG-hphMX6 ade6-M210 leu1-32 ura4-D18	This study
CBL2500	h-	spt16-GFP-kan ade6-M210 leu1-32 ura4-DS/E his3-D1 arg3-D4	Lejeune E *et al* . 2007
CBL2461	h-	spt16-1-kan ade6-M210 leu1-32 ura4-D18	Choi *et al* . 2012
CBL2465	h-	spt16-2-kan ade6-M210 leu1-32 ura4-D18	Choi *et al* . 2012
CBL4056	h-	fft3Δ::hphMX6 spt16-1-kan ade6-M210 leu1-32 ura4-D18	This study
CBL4057	h+	fft3Δ::hphMX6 spt16-1-kan ade6-M210 leu1-32 ura4-D18	This study
CBL4058	h-	fft3Δ::hphMX6 spt16-2-kan ade6-M210 leu1-32 ura4-D18	This study
CBL4059	h+	fft3Δ::hphMX6 spt16-2-kan ade6-M210 leu1-32 ura4-D18	This study
CBL1731	h-	fft3-3HA-TAP-KAN ade6-M210 leu1-32 ura4-D18	This study
CBL474	h-	cph1Δura4 ade6-M210 leu1-32 ura4-D18	This study
CBL3646	h-	fft3Δ::HYG cph1Δ::ura4+ ade6-M210 leu1-32 ura4-D18 #1	This study
CBL3647	h-	fft3Δ::HYG cph1Δ::ura4+ ade6-M210 leu1-32 ura4-D18 #2	This study
CBL3687	h-	fft2Δ::NAT fft3Δ::hph cph1Δ::ura4+ ade6-M210 leu1-32 ura4-D18 #1	This study
CBL3688	h-	fft2Δ::NAT fft3Δ::hph cph1Δ::ura4+ ade6-M210 leu1-32 ura4-D18 #2	This study
CBL3689	h+	fft2Δ::NAT fft3Δ::hph cph1Δ::ura4+ ade6-M210 leu1-32 ura4-D18 #1	This study
CBL3690	h+	fft2Δ::NAT fft3Δ::hph cph1Δ::ura4+ ade6-M210 leu1-32 ura4-D18 #2	This study
CBL1910	h+	pst2Δ::kan ade6-210 his3-D1 leu1-32 ura4-D18 arg3-D4	This study
CBL3794	h+	pst2Δ::KAN fft3Δ::hph ade6-M210 leu1-32 ura4-D18 #1 *	This study
CBL3795	h+	pst2Δ::KAN fft3Δ::hph ade6-M210 leu1-32 ura4-D18 #2 *	This study
CBL1940	h+	h3.1/h4.1Δ::his3+ h3.3/h4.3::arg3+ ura4-D18/DSE leu1-32 arg3-D4 his3-D1	This study
CBL3800	h+	h3.1/h4.1Δ::his3+ h3.3/h4.3::arg3+ ura4-D18/DSE leu1-32 arg3-D4 his3-D1 fft3Δ::hph #1 *	This study
CBL3802	h+	h3.1/h4.1Δ::his3+ h3.3/h4.3::arg3+ ura4-D18/DSE leu1-32 arg3-D4 his3-D1 fft3Δ::hph #3 *	This study

*Genotypes which are relevant to this study are described here.

Supplementary Table 3. Primers used in this study

Name	Descrption	Strand	Sequence
eno101_3ORF_for	3' ORF region at eno101 gene	Fw	CCTTCATCTCCCACTTGA
eno101_3ORF_rev	3' ORF region at eno101 gene	Rev	CGAGTTCCTCCTCGATAC
pyk1_3ORF_for	3' ORF region at pyk1 gene	Fw	CTCAAAGAGCTCGTCAATC
pyk1_3ORF_rev	3' ORF region at pyk1 gene	Rev	GATCTTGTCACCCTTCTTG
